# Transcriptomic and Ectoine Analysis of Halotolerant *Nocardiopsis gilva* YIM 90087^T^ Under Salt Stress

**DOI:** 10.3389/fmicb.2018.00618

**Published:** 2018-03-29

**Authors:** Jian Han, Quan-Xiu Gao, Yong-Guang Zhang, Li Li, Osama A. A. Mohamad, Manik Prabhu Narsing Rao, Min Xiao, Wael N. Hozzein, Dalal H. M. Alkhalifah, Yong Tao, Wen-Jun Li

**Affiliations:** ^1^Key Laboratory of Biogeography and Bioresource in Arid Land, Xinjiang Institute of Ecology and Geography, Chinese Academy of Sciences, Ürümqi, China; ^2^Key Laboratory of Microbial Physiological and Metabolic Engineering, State Key Laboratory of Microbial Resources, Institute of Microbiology, Chinese Academy of Sciences, Beijing, China; ^3^University of Chinese Academy of Sciences, Beijing, China; ^4^Institute for Post Graduate Environmental Studies, Environmental Science Department, Arish University, North Sinai, Egypt; ^5^State Key Laboratory of Biocontrol and Guangdong Provincial Key Laboratory of Plant Resources, School of Life Sciences, Sun Yat-sen University, Guangzhou, China; ^6^Botany and Microbiology Department, Faculty of Science, Beni-Suef University, Beni Suef, Egypt; ^7^Bioproducts Research Chair, Zoology Department, College of Science, King Saud University, Riyadh, Saudi Arabia; ^8^Biology Department, Faculty of Science, Princess Nourah bint Abdulrahman University, Riyadh, Saudi Arabia

**Keywords:** *Nocardiopsis gilva*, halotolerant, ectoine, hydroxyectoine, transcriptomic analysis, whole-cell catalysis

## Abstract

The genus *Nocardiopsis* is an unique actinobacterial group that widely distributed in hypersaline environments. In this study, we investigated the growth conditions, transcriptome analysis, production and accumulation of ectoine by *Nocardiopsis gilva* YIM 90087^T^ under salt stress. The colony color of *N. gilva* YIM 90087^T^ changed from yellow to white under salt stress conditions. Accumulation of ectoine and hydroxyectoine in cells was an efficient way to regulate osmotic pressure. The ectoine synthesis was studied by transferring the related genes (*ectA, ectB*, and *ectC*) to *Escherichia coli*. Transcriptomic analysis showed that the pathways of ABC transporters (ko02010) and glycine, serine, and threonine metabolism (ko00260) played a vital role under salt stress environment. The *ectABC* from *N. gilva* YIM 90087^T^ was activated under the salt stress. Addition of exogenous ectoine and hydroxyectoine were helpful to protect *N. gilva* YIM 90087^T^ from salt stress.

## Introduction

The genus *Nocardiopsis* is an unique actinobacterial group which was first described by Meyer (1976). Members of this genus are widely distributed in hypersaline environments and produce very distinct secondary metabolites (Tsujibo et al., 2003; Sun et al., 2015, 2017; Sharma and Singh, 2016). In past few years, attempts to isolate novel *Nocardiopsis* strains were made and many novel species such as *N. algeriensis* (Bouras et al., 2015), *N. mangrovei* (Huang et al., 2015), *N. oceani* and *N. nanhaiensis* (Pan et al., 2015), *N. ansamitocini* (Zhang et al., 2016b), *N. sediminis* (Muangham et al., 2016), *N. rhizosphaerae* (Zhang et al., 2016c), and *N. akesuensis* (Gao et al., 2016) have been reported. At present, the genus comprises 53 species and 5 subspecies (^1^2017). A large number of *Nocardiopsis* species were isolated from saline or alkaline environments and approximately two-thirds of them were halophilic or halotolerant (Bouras et al., 2015; Pan et al., 2015). To survive in hypersaline environments, *Nocardiopsis* uses various strategies such as reinforcement of cell walls and accumulation of various osmolytes (Ameur et al., 2011; Zhang et al., 2016a). Accumulation of osmolytes play a vital role during salt stress and help in providing osmotic balance without interfering with the essential cellular processes and the normal metabolism (Liu et al., 2017). One of the most abundant osmolytes present in nature is ectoine, which was first found in the halophilic bacterium *Ectothiorhodospira halochloris* (Galinski et al., 1985). Ectoines found to improve protein folding and protect biomolecules such as enzymes, nucleic acids, antibodies, and even whole cells against various stress conditions (Barth et al., 2000).

The next-generation sequencing technologies and whole genome sequencing provide a new perspective for the research (Liu et al., 2017). In the past few years, about 22 genomes of *Nocardiopsis* have been sequenced (^2^2017). Comparative genomic analysis of *Nocardiopsis* species revealed that they retain core genome [at least 2,517 genes (approximately 43% of the genome content)] and also continuously acquire genes and expand their genomes to cope with environmental pressures (Li et al., 2013). Interestingly, the genes related to ectoine biosynthesis were found in genomes of all *Nocardiopsis* species.

Though extensive research was conducted to isolate novel *Nocardiopsis* members and analyzing their genome, yet only few reports focused on understanding mechanism for their survival under salt stress.

The objectives of the present study were: (1) compare the growth status of *Nocardiopsis gilva* YIM 90087^T^ under different saline conditions. (2) Analyze intracellular and extracellular concentrations of ectoine and hydroxyectoine under different salt stress. (3) Evaluate the mRNA expressions of the genes related to ectoine biosynthesis by RNA-seq sequencing under different salt stress. (4) Study the effects of ectoine and hydroxyectoine on the growth of *N. gilva* YIM 90087^T^ in presence and absence of salt stress. (5) Investigate the effects of saline conditions on ectoine biosynthesis of *N. gilva* YIM 90087^T^, by reconstructing its biosynthetic pathway and studying the process of ectoine biosynthesis in *E. coli* BW25113. (6) Identify the ectoine from recombinant *E. coli* using liquid chromatography–mass spectrometry (LC–MS).

## Materials and Methods

### Strains and Media

*Nocardiopsis gilva* YIM 90087^T^, a halotolerant actinobacterium, used in this study was isolated by one of our group members from hypersaline soil in Xinjiang Province, China (Li et al., 2006). *E. coli* DH5α, and BW25113 [F^-^, *Δ*(*araD-araB*)*567, ΔlacZ4787*(::rrnB-3), *λ*^-^, *rph-1, Δ*(*rhaD-rhaB*)*568, hsdR514*] were used as the host strains for the construction of recombinant plasmids and ectoine biosynthesis, respectively.

2YT medium composed of (L^-1^) 16 g tryptone and 10 g yeast extract. Luria-Bertani (LB) medium composed of (L^-1^) 10 g NaCl, 10 g tryptone, and 5 g yeast extract. ZYM medium composed of (L^-1^) 10 g tryptone, 5 g yeast extract, 5 g glycerol, 0.5 g glucose, 25 mmol Na_2_HPO_4_, 50 mmol NH_4_Cl, 25 mmol KH_2_PO_4_, 5 mmol Na_2_SO_4_, 2 mmol MgSO_4_, 50 mmol FeCl_2_, 0.02 mmol CaCl_2_, 0.01 mmol MgCl_2_, 0.01 mmol ZnSO_4_, 0.002 mmol CoCl_2_, 0.002 mmol CuCl_2_, 0.002 mmol NiCl_2_, 0.002 mmol Na_2_MoO_4_, 0.002 mmol Na_2_SeO_3_, 0.002 mmol H_3_BO_3_, and 0.06 mmol HCl. EB buffer consisted of 100 mM sodium aspartate, 100 mM glutamate, 100 mM glycerol, 100 mM KCl, and 100 mM sodium phosphate buffer (pH 7.0).

### Effect of Salt Concentrations on Colony Morphology, Growth, and Ectoine Biosynthesis of *N. gilva* YIM 90087^T^

To investigate the effects of NaCl concentrations on the growth of *N. gilva* YIM 90087^T^, 1 ml of culture was inoculated in 100 ml of 2YT medium having NaCl concentration ranging 0–15% w/v (with an increment of 5%). The flasks were incubated at 30°C for 96 h. The growth was estimated by measuring the optical density at 600 nm (OD_600_). The variations of colony morphology under different NaCl stress were evaluated using 2YT agar at 30°C for 96 h. To study the effects of NaCl concentartions on exogenous ectoine and hydroxyectoine, 1 mg/ml of ectoine and hydroxyectoine were added to 2YT medium. *N. gilva* YIM 90087^T^ grew in these media under different salt concentartions at 30°C for 96 h.

### Molecular Methods

Genomic DNA from *N. gilva* YIM 90087^T^ was extracted and purified using the Bacterial DNA Kit (Omega Bio-Tek, United States). Plasmid DNA was extracted and purified from *E. coli* DH5α using the Plasmid Mini Kit I (Omega Bio-Tek). Restriction endonucleases, T4 DNA ligase, and Q5 High-Fidelity DNA polymerase (New England Biolabs, United States) were used according to the manufacturer’s instructions. Standard methods were used for transformation (Chong, 2001).

*NgiectA* and *NgiectBC* were amplified from the genomic DNA of *N. gilva* YIM 90087^T^ (NCBI Reference Sequence: NZ_ANBG01000167.1) by using PCR primers NgiectA-F/R and NgiectBC-F/R as follows: NgiectA-F: 5′-CATGCCATGGGCTCCCGTGATAATTCCTCCC-3′, NgiectA-R: 5′-CGGGATCCTCAGTTCCGGCCGCTGACCGCCGAC-3′; NgiectBC-F: 5′-CGGGATCC*AAGGAG*ATATACATGGAGATCTTCGACCGCCTCG-3′, NgiectBC-R: 5′-GGAATTCCTACTCGGCCGTGGCCAGGCTCTCC-3′ (Restriction enzyme cutting site were underlined, and ribosome bind site were in italicized). The *NgiectA* PCR product was digested with *Nco* I and *Bam*H I, while *NgiectBC* PCR product was digested with *Bam*H I and *Eco*R I. The two DNA fragments were inserted together into the sites of *Nco* I and *Eco*R I in pBAD-HisB (Invitrogen, United States) resulting in the plasmid pBNABC. The vector pBNABC was transformed into *E. coli* BW25113 (named pBNABC/BW25113).

The total RNA from YIM 90087^T^ (culture of mid-log growth phase) was extracted using RNAprep Pure Cell/Bacteria Kit (TIANGEN, CN). RNA concentration was measured by UVS-99 (ActGene, United States). RNA quality was assessed by electrophoresis on a denaturing agarose gel. The samples of transcription were sequenced and analyzed by Biomarker Technologies, China.

### Ectoine Biosynthesis in Recombinant *E. coli*

Ectoine biosynthesis was performed using the method of whole-cell catalysis in *E. coli* pBNABC/BW25113 (He et al., 2015). To obtain ectoine producer, *E. coli* pBNABC/BW25113 was grown in ZYM medium with ampicillin by adding 2 g/L L-arabinose as an inducer at 30°C, 200 rpm for 16 h. The induced cells were harvested by centrifugation (6000 × *g*, 20 min) and washed with 0.9% NaCl buffer twice. The cells were re-suspended with 50 ml of EB buffer (OD_600 nm_ = 10). The catalysts mixtures were shaken in a 250-ml flask at 30°C, 200 rpm for 96 h.

### HPLC and LC–MS Analysis of Ectoine and Hydroxyectoine

To identify ectoine and hydroxyectoine, 1 ml of cells suspension was harvested by centrifugation and extracted according to the method of Bligh and Dyer (1959). The intracellular and extracellular concentrations of ectoine and hydroxyectoine were measured by isocratic HPLC (Agilent 1260 series, Hewlett-Packard) following the method of He et al. (2015). The ectoine from recombinant *E. coli* was identified using Agilent 1260/6460 high-performance liquid chromatography/triple quadrupole mass spectrometer (Agilent, United States) using ESI source in positive ionization (He et al., 2015).

## Results

### Effect of Salt Concentrations on Colony Morphology and Growth of *N. gilva* YIM 90087^T^

**FIGURE 1:**
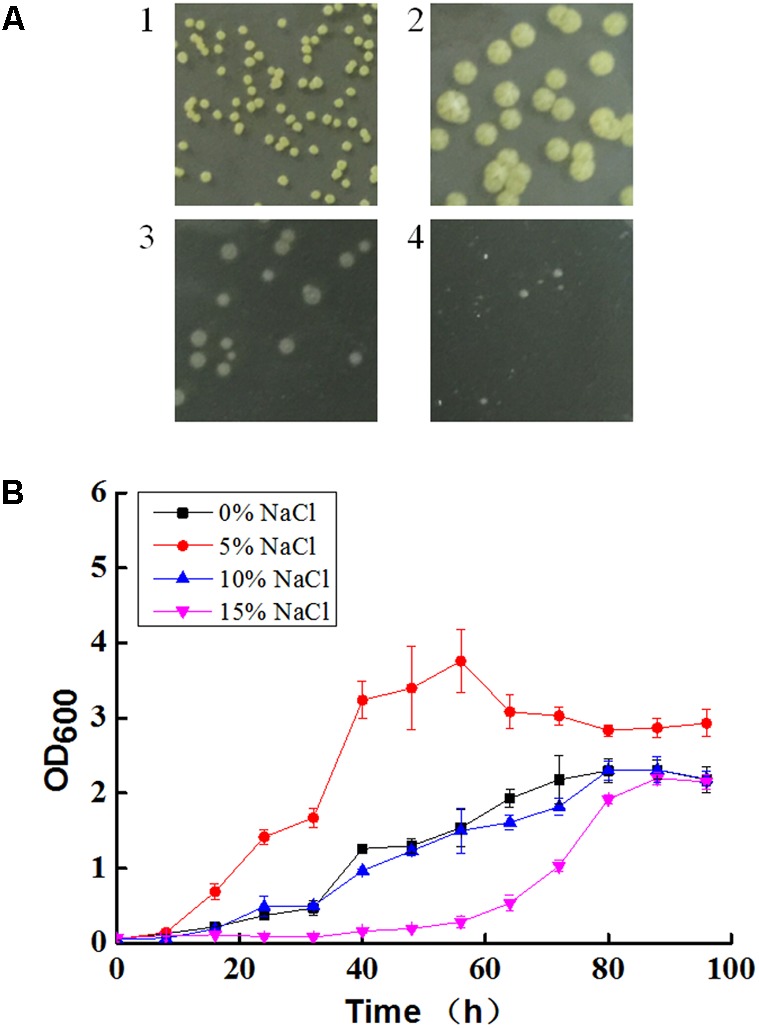
Colony morphology **(A)** and growth curve **(B)** of *Nocardiopsis gilva* YIM 90087^T^ with varying concentrations of NaCl. A1: without NaCl; A2: 5% NaCl; A3: 10% NaCl; A4: 15% NaCl.

The colony color of *N. gilva* YIM 90087^T^ changed from yellow to white with the increase of salt concentration (**Figure 1A**). *N. gilva* YIM 90087^T^ had the optimum growth at 5% NaCl concentration and the growth rate declined with the increase or decrease of the NaCl concentration (**Figure 1B**).

### Differential Expression Analysis

**FIGURE 2:**
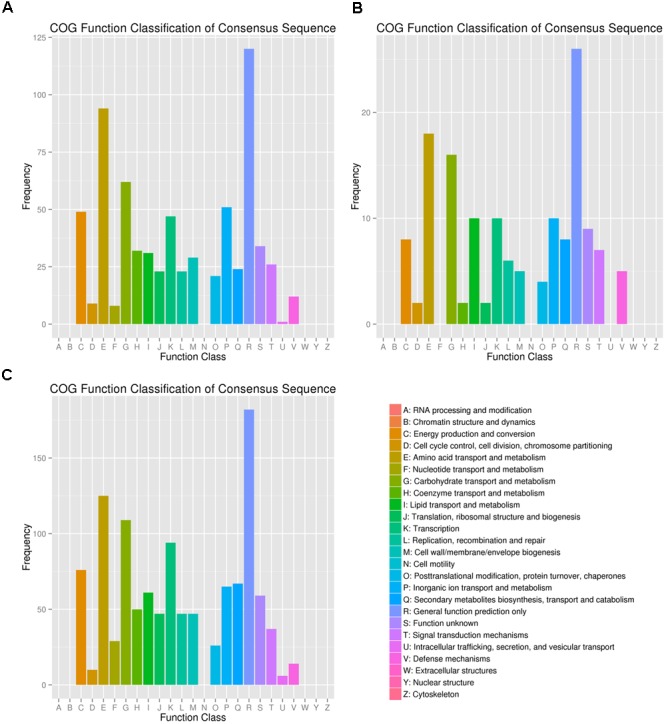
COG classifications of the three DEG sets. The DEGs from the three sets (**A**: 5%/0%; **B**: 10%/0%; **C**: 15%/0%) had COG classifications among the 25 groups. The capital letters on the x-axis indicates the COG groups as listed on the right of the histogram. The y-axis indicates the number of DEGs.

**FIGURE 3:**
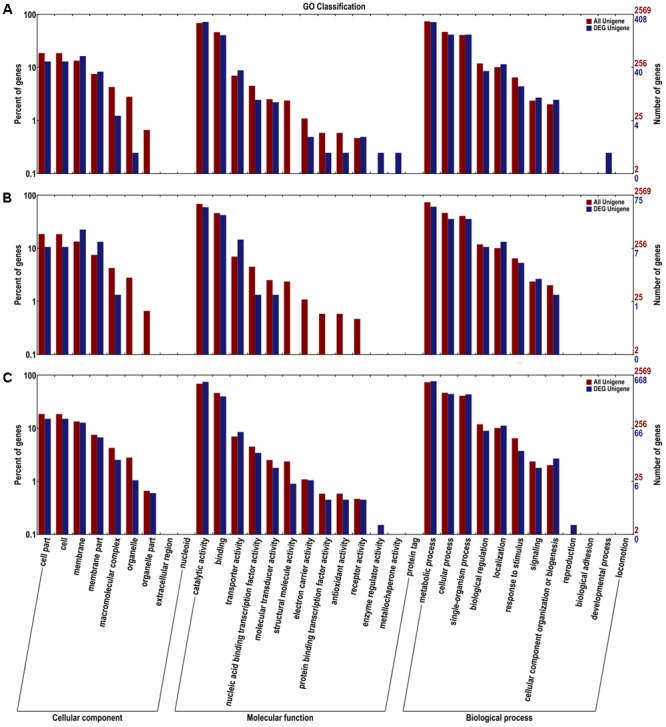
Gene ontology (GO) terms enrichments of the DEGs from the three sets. The DEGs were divided into three clusters: cellular component, molecular function, and biological process. **(A)** 5%/0%; **(B)** 10%/0%; **(C)** 15%/0%. The right y-axis presents the number of unigenes in the clusters, while the left y-axis presents the percentage of a specific category of unigenes in that clusters.

FDR < 0.01 and Fold Change ≥ 2 were used as the selection criteria to identify the differentially expressed gene (DEG). The DEGs were annotated in COG, GO, KEGG, Swissport, and NR databases. The numbers of DEGs, up-regulated and down-regulated genes, and annotated DEGs were mentioned in **Table 1**. COG classifications of the three DEG sets were presented in 25 COG groups (**Figure 2**). Amino acid transport and metabolism and carbohydrate transport and metabolism were the dominant groups in all the three DEG sets. DEGs were distributed to 34 GO groups, which constitute three domains: cellular component, molecular function, and biological process (**Figure 3**). The DEGs from the three sets (5%/0%, 10%/0%, and 15%/0%) were mapped to 77, 32, and 90 reference canonical pathways in KEGG database respectively. The DEGs number and enrichment analysis were mentioned in **Table 2**. The DEGs number and corrected *P*-values results indicated that DEGs in the pathways of ABC transporters (ko02010) and glycine, serine, and threonine metabolism (ko00260) play extremely significant roles under salt stress. Particularly in maximum-tolerated NaCl concentrations, expressions of ABC transporters genes of ectoine/hydroxyectoine and glycine betaine, and ectoine/hydroxyectoine synthesis genes increased evidently.

**Table 1:** Statistics of DEGs and annotations.

	DEGs set		
	5%/0%	10%/0%	15%/0%
DEG number	1026	231	1578
Up-regulated	493	62	621
Down-regulated	533	169	957
COG	500	102	796
GO	408	75	668
KEGG	300	58	510
Swissprot	797	151	1219
NR	977	207	1470

**Table 2:** The DEG number, proportion, and *Q*-value in the pathway.

DEGs sets	Pathway number	Function	DEG number	Proportion (%)	Corrected *P*-value
5%/0%	ko02010	ABC transporters	37	21.14	0.02
	ko00260	Glycine, serine, and threonine metabolism	14	8.00	0.2
	ko00650	Butanoate metabolism	14	8.00	0.48
	ko00230	Purine metabolism	12	6.86	1
	ko02020	Two-component system	9	5.14	1
	ko00280	Valine, leucine, and isoleucine degradation	9	5.14	1
	ko00071	Fatty acid metabolism	9	5.14	1
	ko00362	Benzoate degradation	9	5.14	1
	ko00190	Oxidative phosphorylation	8	4.57	1
	ko00290	Valine, leucine, and isoleucine biosynthesis	8	4.57	1
	ko00500	Starch and sucrose metabolism	8	4.57	0.96
10%/0%	ko02010	ABC transporters	8	29.63	0.42
	ko00500	Starch and sucrose metabolism	3	11.11	0.42
	ko00260	Glycine, serine, and threonine metabolism	2	7.41	1
15%/0%	ko02010	ABC transporters	34	10.4	1
	ko00260	Glycine, serine, and threonine metabolism	22	6.73	0.09
	ko00230	Purine metabolism	18	5.5	1
	ko00240	Pyrimidine metabolism	17	5.2	1
	ko02020	Two-component system	16	4.89	1
	ko00010	Glycolysis/gluconeogenesis	15	4.59	1
	ko00860	Porphyrin and chlorophyll metabolism	15	4.59	1
	ko00620	Pyruvate metabolism	14	4.28	1
	ko00627	Aminobenzoate degradation	14	4.28	1
	ko00650	Butanoate metabolism	12	3.67	1
	ko00020	Citrate cycle (TCA cycle)	12	3.67	1
	ko00030	Pentose phosphate pathway	12	3.67	0.86
	ko00362	Benzoate degradation	12	3.67	1
	ko00562	Inositol phosphate metabolism	10	3.06	0.29
	ko02060	Phosphotransferase system	7	2.14	0.53

### Genetic Organization of *ect* Gene Cluster in *N. gilva* YIM 90087^T^

**FIGURE 4:**
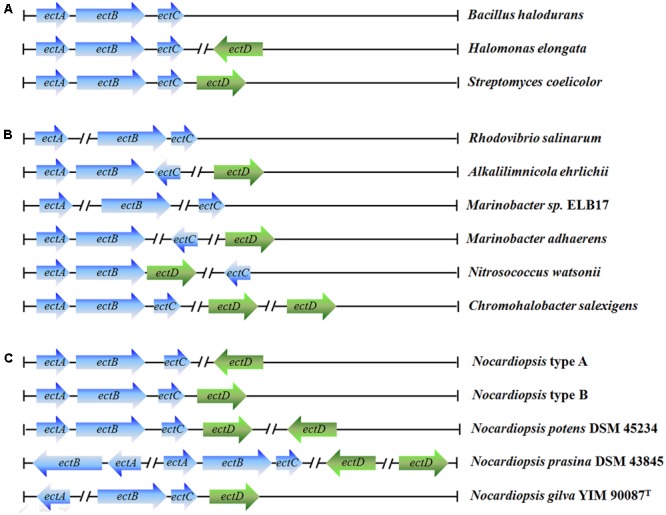
Genetic organization of the ectoine/hydroxyectoine biosynthesis gene clusters. The different types of ectoine and hydroxyectoine biosynthesis gene clusters present in microorganisms: **(A)** most common organizational types of the ect gene clusters and representative strains; **(B)** unusual types of the ect gene clusters and representative strains; **(C)** different types of the ect gene clusters from 21 *Nocardiopsis* strains.

We analyzed synthesis genes of ectoine and hydroxyectoine in *Nocardiopsis*. The *ectABCD* (synthesis genes) was found in *Nocardiopsis* genome. There are two common organizational types of *ectABCD* gene cluster on the 18 whole-genome sequences of *Nocardiopsis* strains (**Figure 4**). The *ectABC* were belonging to a gene cluster, and the *ectD* distributes on other locations of the genome (type A, 15 strains) or the *ectABCD* belongs to the same gene cluster (type B, 3 strains). Additionally, more than one *ectAB* or *ectD* genes were found in *N. potens* DSM 45234 and *N. prasina* DSM 43845. However, the organizational types of *ect* gene cluster from *N. gilva* YIM 90087^T^ was clearly different from others *Nocardiopsis* strains. The *ectA* gene completely separated from *ectBCD* gene cluster, which deviate from the commonly found genetic organization. Moreover, the *ehuABCD*, ectoine/hydroxyectoine ABC transporter gene, were situated on the downstream of *ectD* gene in genome.

### Expression of Genes Involved in Synthesis and Transport of Ectoine and Hydroxyectoine

We analyzed the mRNA expression of the genes involved in ectoine/hydroxyectoine synthesis and transport at different salt concentrations (**Table 3**). The genes involved in ectoine biosynthesis process presented different levels of up-regulation with the increase of NaCl concentration, particularly in 15% NaCl. Unlike other genes, *ectD* presented two stepwise up-regulations from 5 to 10% NaCl concentration and from 10 to 15% NaCl concentration. Two potential ABC transporter genes (*ehuABCD* and *proXWV*) involved in transport of ectoine were found in *N. gilva* YIM 90087^T^. The mRNA expressions of them varied with increase NaCl concentration. The expressions of *ehuABCD* were positively correlated with NaCl concentration when the NaCl concentration was above 5% in the medium. Yet, the expressions of *proXWV* had no obvious changes in optimum and mild-tolerated NaCl concentrations. Interestingly, *ehuABCD* and *proXWV* were activated strongly at maximum-tolerated NaCl concentrations.

**Table 3:** The fold changes of the genes involved in ectoine and hydroxyectoine synthesis and transport in response to different salt stress.

Unigene ID	Genes	Function	Fold change		
			5%/0%	10%/0%	15%/0%
GL001695	*ask*	Aspartate kinase	1.29	1.51	4.45
GL001694	*asd*	L-Aspartate-beta-semialdehyde-dehydrogenase	1.42	1.32	4.16
GL003065	*ectB*	Diaminobutyrate-2-oxoglutarate transaminase	2.16	1.84	7.35
GL001325	*ectA*	Diaminobutyrate acetyltransferase	1.33	2.23	5.26
GL003066	*ectC*	Ectoine synthase	2.58	2.22	7.59
GL003067	*ectD*	Ectoine hydroxylase	2.60	5.91	22.94
GL003068	*ehuB*	Ectoine/hydroxyectoine ABC transporter substrate-binding protein	1.17	1.73	5.85
GL003069	*ehuC*	Ectoine/hydroxyectoine ABC transporter permease subunit	0.88	2.67	10.48
GL003070	*ehuD*	Ectoine/hydroxyectoine ABC transporter permease subunit	1.19	1.95	8.48
GL003071	*ehuA*	Ectoine/hydroxyectoine ABC transporter ATP-binding protein	0.85	2.262	7.18
GL003184	*proX*	Glycine betaine/proline transport system substrate-binding protein	0.80	0.90	4.07
GL003185	*proX*	Glycine betaine/proline transport system substrate-binding protein	1.00	1.28	5.31
GL003186	*proW*	Glycine betaine/proline transport system permease protein	0.90	1.57	9.49
GL003187	*proV*	Glycine betaine/proline transport system ATP-binding protein	0.85	1.47	10.61

### Accumulation of Ectoine and Hydroxyectoine in *N. gilva* YIM 90087^T^

**FIGURE 5:**
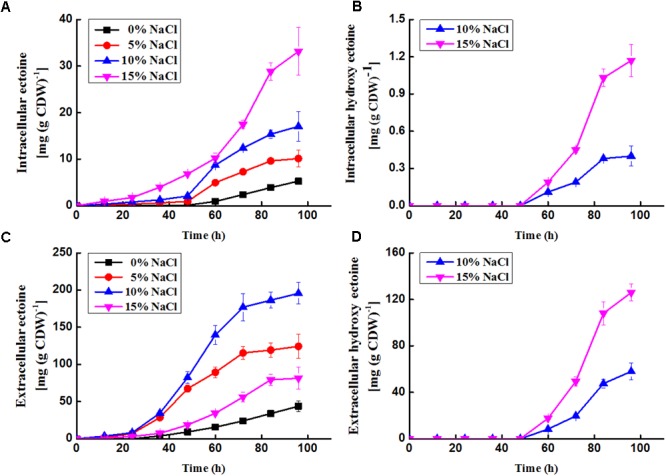
Time profiles of production of ectoine and hydroxyectoine in *N. gilva*. **(A)** Intercellular concentration of ectoine; **(B)** intercellular concentration of hydroxyectoine; **(C)** extracellular concentration of ectoine; **(D)** extracellular concentration of hydroxyectoine. 1 ml of the cells (*N. gilva* YIM 90087^T^, OD_600_ = 1) is about 0.00063 g (DCW).

Ectoine and hydroxyectoine were analyzed by HPLC. The results showed that there were two peaks with the same retention time as the two standards [ectoine (816189, Sigma-Aldrich) and hydroxyectoine (70709, Sigma-Aldrich)] appeared from *N. gilva* YIM 90087^T^ cell. As shown in **Figures 5A,B**, the intracellular concentration changes of ectoine and hydroxyectoine were a cumulative progress. Whereas accumulation of ectoine and hydroxyectoine were influenced by NaCl concentration. They were positively correlated with increasing NaCl concentration. Noteworthy, hydroxyectoine were difficult to be synthesized in the medium with 0 or 5% NaCl. After 48 h, hydroxyectoine were detected in 2YT medium with 10 and 15% NaCl. Moreover, the amounts of ectoine and hydroxyectoine were increased with the increasing cultivation time. After 96 h, the highest level of ectoine (33.14 mg/g CDW) and hydroxyectoine (1.17 mg/g CDW) were detected in 15% NaCl concentration. The ectoine concentrations were 42.6 and 28.3 times higher than hydroxyectoine in 10 and 15% NaCl concentration, respectively. Therefore, the results indicates that ectoine was accumulated in *N. gilva* YIM 90087^T^ cells.

On the other side, we detected ectoine and hydroxyectoine outside the cells (**Figures 5C,D**). A large proportion of ectoine was released out of the cells. After 96 h, 89.20, 92.46, 91.99, and 71.09% of ectoine were released in the medium with 0, 5, 10, and 15% NaCl, respectively. About 99.31 and 99.08% of hydroxyectoine found out of the cells in medium with 10 and 15% NaCl, respectively. The transfer capacity for ectoine enhanced remarkably in the medium with 15% NaCl. Yet, the capacity for hydroxyectoine had no obvious change as concentration added. In our study, we observed the transformation from ectoine to hydroxyectoine with the increase of NaCl concentration. About 50% ectoine transformed into hydroxyectoine at 15% NaCl concentration. The highest extracellular amount of ectoine and hydroxyectoine were found at 10 and 15% NaCl concentration, respectively.

### Effect of Exogenous Ectoine and Hydroxyectoine on Growth of *N. gilva* YIM 90087^T^

**FIGURE 6:**
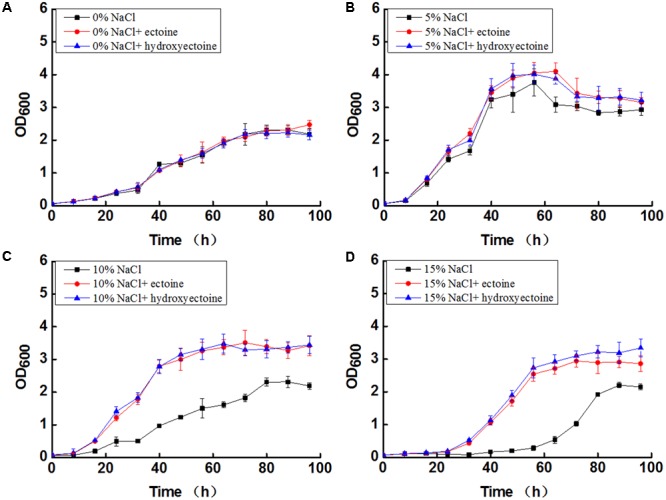
The effect of ectoine and hydroxyectoine on Growth of *N. gilva* YIM 90087^T^ with varying concentrations of NaCl. **(A)** Curve of growth in the mediums with 0% NaCl; **(B)** curve of growth in the mediums with 5% NaCl; **(C)** curve of growth in the mediums with 10% NaCl; **(D)** curve of growth in the mediums with 15% NaCl.

Exogenous ectoine and hydroxyectoine under salt stress increased the growth rate of *N. gilva* YIM 90087^T^. The growth of *N. gilva* YIM 90087^T^ in absence of NaCl was basically the same, in presence or absence of exogenous ectoine and hydroxyectoine (**Figure 6A**). However, with the increase of NaCl concentrations, exogenous ectoine, and hydroxyectoine promoted the growth (**Figure 6**). The addition of ectoine and hydroxyectoine under optimum NaCl concentration of (5%) slightly increased the OD_600_ values when compared in their absence (**Figure 6B**). The addition of ectoine and hydroxyectoine at high NaCl concentration (10–15%) drastically increased the OD_600_ values when compared in their absence (**Figures 6C,D**). It was also noted that the growth with hydroxyectoine was somewhat better than ectoine at 15% NaCl concentration.

### Accumulation of Ectoine in *E. coli*

**FIGURE 7:**
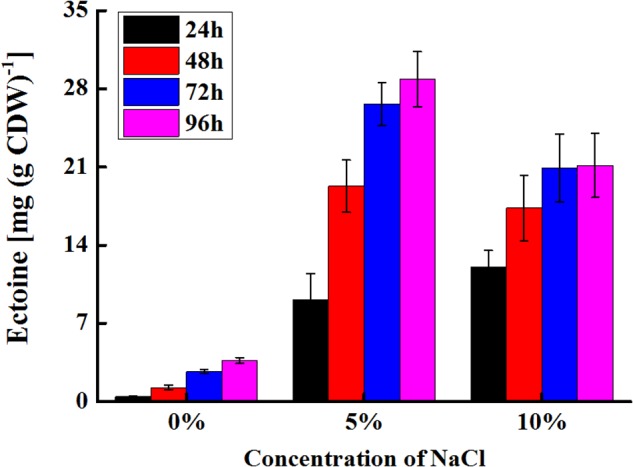
Production changes of ectoine in *E. coli*. After induced, recombinant *E. coli* was shaken in ectoine biosynthesis buffer with different NaCl (0–15%). The ectoine productions were measured every 24 h. Ectoine did not detect in the buffer with 15% NaCl. 1 ml of the cells (*E. coli* pBNABC/BW25113, OD_600_ = 1) is about 0.00034 g (DCW).

Using aspartate as a substrate, glutamate as amino donor and glycerol as an acetyl-CoA donor, ectoine was produced by *E. coli* pBNABC/BW25113. The changes in the production of ectoine were shown in **Figure 7**. Ectoine was produced hardly at 15% NaCl buffer, and a few of ectoine was detected in 0% NaCl buffer. The synthetic rate of ectoine dropped significantly after 24 and 48 h at 10 and 5% NaCl buffer, respectively. The maximum values of initial synthetic rate [0.5 mg (g CDW)^-1^ h^-1^] and final output [28.85 mg (g CDW)^-1^] were found in 10 and 5% NaCl buffer, respectively.

## Discussion

Halophilic microorganisms have the ability to adjust saline environments more quickly and efficiently (Liu et al., 2017). Survival and growth of microorganisms in saline environments require numerous morphological ecotypes and adaptations (Kralj Kuncic et al., 2010; Liu et al., 2017). Salt stress causes change in colony morphology especially, colony color hence, in the present study colony color under salt stress was evaluated. The colony color of *N. gilva* YIM 90087^T^ changed from yellow to white with the increase of salt concentration. Similar result was observed in many halophilic microorganisms when grown in high salt stress (Kralj Kuncic et al., 2010; Liu et al., 2017). Compatible solutes play significant roles to cope with hyperosmotic stress (Poolman and Glaasker, 1998; Ventosa et al., 1998; Roesser and Muller, 2001). These compatible solutes includes sugars (sucrose, trehalose), polyols (glycerol, sorbitol, mannitol, α-glucosylglycerol, mannosyl-glycerol, and mannosyl-glyceramide), *N*-acetylated diamino acids (*N*-acetylglutaminylglutamine amide), betaines (glycine betaine and derivatives), amino acids (proline, glutamate, glutamine, and alanine), and ectoine (hydroxyectoine) (Pastor et al., 2010). Halophilic microorganisms can synthesize different compatible solutes according to the external conditions, such as duration of the osmotic stress, level of salinity, availability of substrates, and osmolytes in the surroundings (Roberts, 2005; Aston and Peyton, 2007; Canamas et al., 2007). *Nocardiopsis* is a special halophilic microorganism, which can synthesize a variety of compatible solutes, such as ectoine, hydroxyectoine, trehalose, glutamate and β-glutamate and so on (Pastor et al., 2010). The related genes involved in compatible solutes synthesis were found in most of the *Nocardiopsis* species genomes (Li et al., 2013).

In *N. gilva* YIM 90087^T^, the transcriptional regulation was influenced by NaCl stress. We found that ABC transports and glycine, serine, and threonine metabolism pathway were involved in essential biological processes in response to salinity (**Table 3**). The ProP and ProU systems (Aliases of ProXWV) involved in uptake and accumulation of ectoine, proline, and glycine betaine in *E. coli* (Jebbar et al., 1992). The EhuABCD, whose expression is induced by ectoine, specifically facilitates the movement of ectoine and hydroxyectoine across the membrane in *Sinorhizobium meliloti* (Jebbar et al., 2005). The increase of expression levels of these proteins with the increase of NaCl concentration were also found in *N. xinjiangensis* (Zhang et al., 2016a). Glycine betaine and ectoine/hydroxyectoine were two types of significant compatible solutes in glycine, serine, and threonine metabolism pathway. They offered effective protection for cells against salinity, but we found that only expressions of ectoine and hydroxyectoine synthesis genes were positively correlated with increasing of NaCl concentration in *N. gilva* YIM 90087^T^.

Ectoine and hydroxyectoine are extremely vital compatible solute. Addition of ectoine and hydroxyectoine was helpful for the growth of *N. gilva* YIM 90087^T^ at maximum-tolerated NaCl concentrations. Ectoine and hydroxyectoine showed similar effect in *Virgibacillus pantothenticus* in response to high salinity (Kuhlmann et al., 2011). Besides this, ectoine and hydroxyectoine serve as protectants for proteins (Lippert and Galinski, 1992), nucleic acids (Malin et al., 1999), whole cell (Manzanera et al., 2002, 2004a,b), and skin (Heinrich et al., 2007).

Like many halophilic microorganisms (Kuhlmann and Bremer, 2002; Zhang et al., 2009), the synthesis and accumulation of ectoine and hydroxyectoine were response to NaCl stress in *N. gilva* YIM 90087^T^. Transcriptional regulation in response to salinity was paramount. The *ectABCD* genes present different degrees of up-regulation in the range from 5 to 15% NaCl concentration, particularly at 15% NaCl concentration. Ectoine was converted into hydroxyectoine because of higher expression of *ectD*. Intracellular hydroxyectoine was far lower than ectoine in *N. gilva* YIM 90087^T^. It was inclined to use ectoine as compatible solutes in cell. However, in *Prauserella alba*, a moderately halophile from saline soil (Li et al., 2003), hydroxyectoine replaced ectoine and became main compatible solute in the cell, when the salt concentration reached up to 15% outside the cell (Li et al., 2011).

**FIGURE 8:**
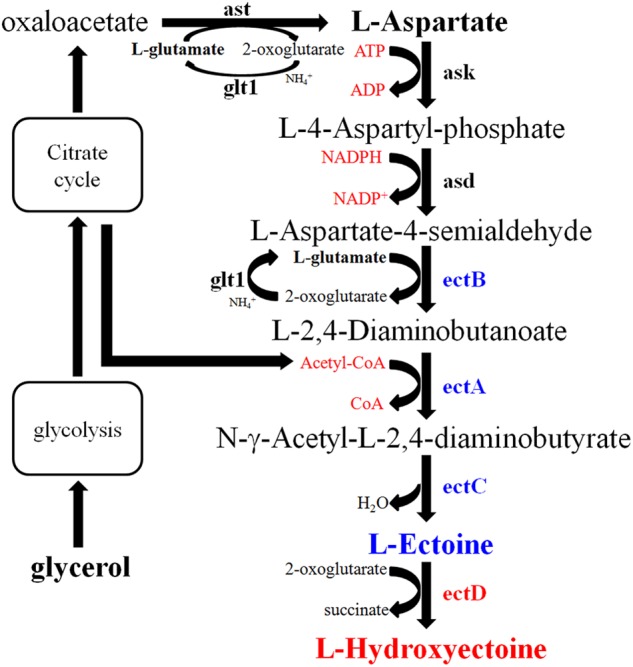
Biosynthetic pathway of ectoine/hydroxyectoine. Ast, aspartate transaminase; Glt1, glutamate synthase; Ask, aspartate kinase; Asd, L-aspartate-beta-semialdehyde-dehydrogenase; ectB, diaminobutyrate-2-oxoglutarate transaminase; ectA, diaminobutyrate acetyltransferase; ectC, ectoine synthase; ectD, ectoine hydroxylase.

The EctABC activity was also influenced by NaCl concentration. To study this effect, *E. coli* pBNABC/BW25113 was constructed. The expression level of EctABC was completely consistent in cell after induction by arabinose. An equal number of the cells were provided in the process of whole-cell catalysis. The aspartate was catalyzed by Ask (aspartate kinase) and Asd (L-aspartate-beta-semialdehyde dehydrogenase) from *E. coli, ectB* (diaminobutyrate-2-oxoglutarate transaminase), *ectA* (diaminobutyrate acetyltransferase), and *ectC* (ectoine synthase) from *N. gilva*, and finally transformed into ectoine (**Figure 8**). This way, *E. coli* could be used to produce ectoine under high-salt conditions, if the related enzymes were not inactive (Lin et al., 2015). Unlike the EctABC from *Halomonas elongata* (He et al., 2015), EctABC from *N. gilva* YIM 90087^T^ were activated under the salt stress in *E. coli*. However, the biosynthesis of ectoine was completely blocked at 15% NaCl concentration. The metabolism of host cell was inhibited in high salt environment (Metris et al., 2014). The enzymes from the host *E. coli*, such as Ask, Asd, and the enzymes involved in transamination and synthesis of acetyl-CoA, tend to keep the activity at the lower salt environments.

## Conclusion

In this study, we analyzed various response of *N. gilva* YIM 90087^T^ at different NaCl concentration. Under salt stress, *N. gilva* YIM 90087^T^ colony color changed. The addition of ectoine and hydroxyectoine provided protection for *N. gilva* YIM 90087^T^ at high salt environment. Transcriptomic analysis of *N. gilva* YIM 90087^T^ indicated that, the synthesis and accumulation of ectoine and hydroxyectoine was an effective way to regulate osmotic pressure. Internal and external ectoine and hydroxyectoine analysis showed that synthesis of ectoine and hydroxyectoine raised with increase of NaCl concentration. Ectoine was transformed into hydroxyectoine gradually, however, ectoine was still the dominant compatible solute in the cell. To study the activity, *ectABC* from *N. gilva* YIM 90087^T^ transferred into *E. coli* BW25113. At the same expression level, we study the activity of *ectABC* with different NaCl concentration, and found that they were activated with a certain NaCl concentrations.

## Author Contributions

W-JL, YT, DA, and JH designed the research and project outline. JH, Y-GZ, and OM performed the growth and morphology observation. JH, Y-GZ, LL, and MN performed the transcriptome sample preparation and sequencing. JH, Q-XG, and W-JL provided HPLC and LC–MS analysis of ectoine and hydroxyectoine. JH, MN, MX, WH, YT, and W-JL drafted the manuscript. All authors read and approved the final manuscript.

## Conflict of Interest Statement

The authors declare that the research was conducted in the absence of any commercial or financial relationships that could be construed as a potential conflict of interest.

## Footnotes

1 http://www.bacterio.net/nocardiopsis.html

2 https://www.ncbi.nlm.nih.gov/genome
